# Quantitative proteome-wide O-glycoproteomics analysis with FragPipe

**DOI:** 10.1007/s00216-024-05382-x

**Published:** 2024-06-15

**Authors:** Daniel A. Polasky, Lei Lu, Fengchao Yu, Kai Li, Michael R. Shortreed, Lloyd M. Smith, Alexey I. Nesvizhskii

**Affiliations:** 1 Department of Pathology, University of Michigan, Ann Arbor, MI USA; 2 Department of Chemistry, University of Wisconsin-Madison, Madison, WI USA; 3 Department of Pharmaceutical Chemistry, University of San Francisco, San Francisco, CA USA; 4 Department of Computational Medicine and Bioinformatics, University of Michigan, Ann Arbor, MI USA

**Keywords:** Glycoproteomics, Software, O-Glycopeptides, Localization

## Abstract

Identification of O-glycopeptides from tandem mass spectrometry data is complicated by the near complete dissociation of O-glycans from the peptide during collisional activation and by the combinatorial explosion of possible glycoforms when glycans are retained intact in electron-based activation. The recent O-Pair search method provides an elegant solution to these problems, using a collisional activation scan to identify the peptide sequence and total glycan mass, and a follow-up electron-based activation scan to localize the glycosite(s) using a graph-based algorithm in a reduced search space. Our previous O-glycoproteomics methods with MSFragger-Glyco allowed for extremely fast and sensitive identification of O-glycopeptides from collisional activation data but had limited support for site localization of glycans and quantification of glycopeptides. Here, we report an improved pipeline for O-glycoproteomics analysis that provides proteome-wide, site-specific, quantitative results by incorporating the O-Pair method as a module within FragPipe. In addition to improved search speed and sensitivity, we add flexible options for oxonium ion-based filtering of glycans and support for a variety of MS acquisition methods and provide a comparison between all software tools currently capable of O-glycosite localization in proteome-wide searches.

**Graphical Abstract:**

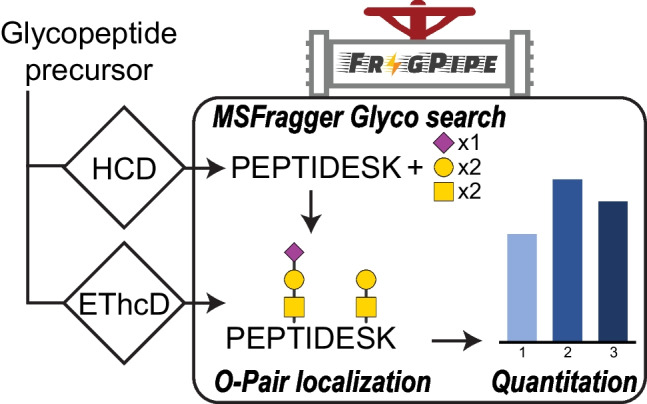

**Supplementary Information:**

The online version contains supplementary material available at 10.1007/s00216-024-05382-x.

## Introduction

Glycosylation of proteins is an abundant post-translational modification with numerous roles in biology and disease [1, 2]. Mass spectrometric analysis of glycopeptides can provide site-specific identification of glycans on individual glycoproteins and throughout the proteome, revealing the “glyco-code” of diseases and transforming our understanding of biology [3–5]. These glycoproteomics methods must overcome numerous challenges, from the macro- and micro-heterogeneity of glycans [3] to the need for enrichment of glycopeptides [6] to complex data acquisition and analysis strategies to account for the lability of glycans in tandem mass spectrometry [7, 8]. O-glycosylation is particularly challenging, as the glycosidic bond between the glycan and peptide is highly labile in positive mode tandem mass spectrometry, typically resulting in complete loss of glycan(s) from the peptide during collisional activation [7, 9]. O-glycans also present challenges in sample preparation and enrichment, recently reviewed elsewhere [6, 7, 10]. Furthermore, mucin-type O-glycosylation [11], an abundant and critical mediator of intercellular and immune interactions [12], often occurs in densely clustered repeating units [13], typically resulting in glycopeptides bearing multiple glycans. Thus, O-glycoproteomics methods must be capable of deconvoluting the mass signatures of multiple glycans into discrete units and localizing them to the correct sites within a glycopeptide.

Identification of O-glycopeptides by software is critical to large-scale O-glycoproteomics but has long been limited by the heterogeneity and lability of O-glycans [8]. Because O-glycans are highly labile during collisional activation, O-glycoproteomics methods have largely utilized electron-based activation methods, such as ETD or EThcD, to generate peptide backbone fragments with the glycan(s) preserved intact, allowing for simultaneous peptide sequencing and glycan localization [10, 14]. Unlike N-glycans, a consensus sequence motif has not been identified for O-glycans. As a result, all serine and threonine residues are typically considered as possible glycosites in database searches. Combined with the heterogeneity of glycans and possibility for multiple glycans to occur within a peptide, this results in a combinatorial explosion of possible glycan localizations. Considering a database of 32 possible glycans, for example, a single glycopeptide can have tens of millions of possible glycan configurations (Table 1). This makes proteome-scale analyses, which need to search millions of peptide sequences, each with their own set of glycan configurations, computationally infeasible for conventional search methods when considering the full O-glycome of an organism. As a result, most current O-glycoproteomic analyses using conventional search tools like Byonic are conducted considering only a small subset of known glycans to reduce the computational burden.

**Table 1:** Example of combinatorial explosion of possible glycan configurations for a single glycopeptide containing varying numbers of possible glycosites. Conventional search enumerates all combinations of all glycans at all sites, whereas peptide-first search enumerates only unique combined glycan masses

Glycan database size	Glycosites in peptide	Configurations: conventional search	Configurations: peptide-first search
32	2	528	258
32	5	435,896	6,527
32	8	76,904,684	38,370

We have previously developed the MSFragger [15] search engine and FragPipe environment for ultrafast proteomics searches in a variety of contexts, including glycoproteomics [16–18]. Because all glycans typically dissociate from the peptide during the collisional scan, an open or mass offset search can readily identify the peptide by searching for backbone fragments with no glycans and allowing a mass difference between the sequence mass of the peptide and the observed precursor mass [15, 18, 19]. This “peptide-first” [8] search strategy considers only unique values for the total mass of all glycans during the initial search, as opposed to all possible configurations of individual glycans at all glycosites, reducing the number of configurations by orders of magnitude for peptides with many glycosites (Table 1). However, for O-glycopeptide analysis, previous FragPipe searches were limited to reporting unlocalized total glycan compositions from collisional activation data or to a conventional search for electron-based activation data. In particular, the lack of localization for O-glycopeptides precluded site-specific quantitative analyses. An elegant solution to this problem was recently developed with the O-Pair search method [20]. It utilizes two paired tandem MS scans of the same glycopeptide precursor, one with collisional activation and the other with electron-based activation, to first identify the peptide sequence and total glycan mass from the collisional scan and then deconvolute and localize the glycan(s) using the electron-based scan and a graph theory-based dynamic programming algorithm. Implemented in the MetaMorpheus search engine [21], O-Pair search was shown to be orders of magnitude faster than conventional searches while also capable of identifying additional glycopeptides due to its divide-and-conquer approach to reducing the total search space [20, 22]. Another glycoproteomics software package, pGlyco3 [23], implemented a similar O-glycosite localization algorithm, with some clever adjustments to account for the glycan-first search of pGlyco vs the peptide-first search of MetaMorpheus (and MSFragger).

Here, we report an improved set of capabilities for O-glycoproteomics in FragPipe, including incorporating O-Pair localization and connecting its output to downstream quantitation tools. We have adapted the original MetaMorpheus O-Pair implementation to be compatible with FragPipe results files and added it as a standalone C# executable within FragPipe that runs within a FragPipe workflow. While the core localization algorithm is largely unchanged, we have added options to analyze a variety of data formats, including single scan EThcD or EAD data in addition to paired scan data, and flexible oxonium ion filtering to improve glycan identifications. Additionally, we show that differences in the initial search methods enable the FragPipe O-Pair method to analyze proteome-wide O-glycoproteomics data with greater speed and to identify additional glycopeptides from scan pairs with low-quality ETD or EThcD spectra. Equipped with quantitation tools for label-free (IonQuant [24]) and isobarically labeled (TMT-Integrator) data, and now including the O-Pair module, FragPipe is capable of performing complete quantitative analyses of N-linked and O-linked DDA glycoproteomics data.

## Materials and methods

### Datasets

All data was downloaded from PRIDE [25] or Massive [26] public repositories via ProteomeXchange [27, 28]. For the “mucin standards” dataset, one raw LC–MS file (2019_09_16_StcEmix_35trig_EThcD25_rep1) was downloaded from repository PXD017646. Briefly, this data consisted of four recombinantly expressed mucin domain-containing proteins digested with StcE [29] and trypsin, analyzed by HCD(35)-pd-EThcD(25) on an Orbitrap mass spectrometer [7]. Numbers in parentheses refer to the normalized collision energy (NCE) or supplemental collision energy for HCD and EThcD/ETciD analyses, respectively. For the “urine” dataset, 10 raw files of wheat germ agglutinin (WGA)-enriched O-glycopeptides from human urine analyzed by HCD(28)-pd-EThcD(15) on an Orbitrap Fusion Lumos instrument were downloaded from MSV000083070. The “GALNT knockout” dataset (PXD036791) contained TMT labeled, enriched O-glycopeptides from N/TERT-1 human skin cell lines with various GalNAc transferases (GALNTs) knocked out, analyzed by HCD(40)-pd-ETciD(25) on an Orbitrap Lumos mass spectrometer [30]. Only samples analyzed on the Lumos instrument were processed here due to differences in acquisition method and data quality to the other data acquired on an Orbitrap Fusion instrument in this repository. The “pGlyco3” dataset refers to inhibitor-initiated homogenous mucin-type O-glycosylation (IMHO) cell lines from the report of Zeng et al. [23], from Massive repository MSV00008677, in which an O-glycan elongation inhibitor was used to prevent the addition of galactose to O-glycans. The O-glycopeptides were analyzed by sceHCD(20/30/40)-pd-EThcD(25) on an Orbitrap Fusion mass spectrometer. Finally, the EThcD-only comparison in Electronic Supplementary Material Figure S1 used 8 EThcD and HCD-pd-EThcD files from repository PXD025407 [31], in which O-glycopeptides enriched by LWAC from HEK293 cells were acquired at a range of supplemental activation energies on an Orbitrap Fusion Lumos instrument.

### O-Pair implementation in FragPipe

A standalone version of O-Pair was extracted from the MetaMorpheus software and set up to run as a C# executable. The standalone O-Pair was modified to translate FragPipe search results from the psm.tsv results table into the MetaMorpheus internal format as input to the O-Pair search. It was further modified to read the mass-calibrated [19] mzML files produced during MSFragger searches to enable using the same calibration for localization as the initial search. An additional oxonium-ion filtering parameter was added to (optionally) prevent matching glycans containing certain monosaccharides to scans lacking defined oxonium ions. If enabled, the oxonium filtering takes a file containing a list of “rules” comprised of pairs of oxonium ion m/z value(s) and associated glycan residue(s). When matching total glycan compositions to the observed glycan mass, a glycan composition containing the residues in a rule is only considered for localization to a given spectrum if the associated oxonium ion(s) in the rule are found in the spectrum with summed relative intensity equal to or greater than the minimum intensity parameter. Oxonium ions are only searched in the first scan of the pair (the collisional activation scan).

**Fig. 1:**
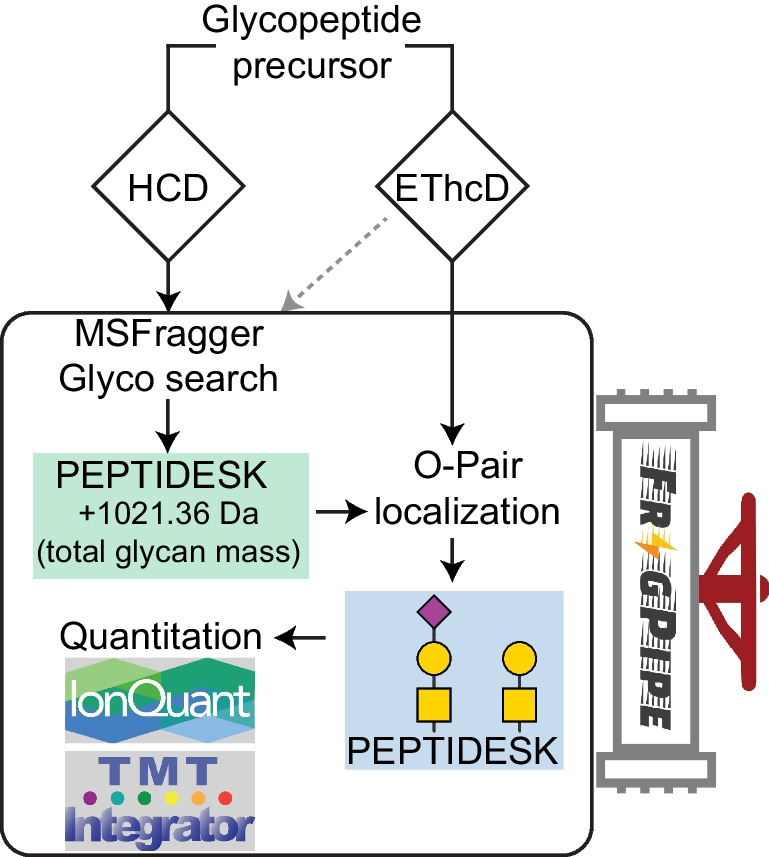
Workflow of O-Pair in FragPipe. Paired MS2 scans of a glycopeptide are analyzed using MSFragger-Glyco search to identify the peptide and total glycan mass from the HCD scan. EthcD-only data can also be searched as an alternative option. O-Pair deconvolutes the total glycan mass to individual localized glycans using the EThcD scan only. O-Pair results are written back to the FragPipe results tables for quantitation

O-Pair search results are written back to the psm.tsv file to be compatible with all downstream quantitation and other tools in FragPipe. Additionally, a utility program was added to FragPipe to pair scans of the same precursor within each raw file to substitute for the parent–child scan pairing performed in MetaMorpheus. The FragPipe scan pairing program considers only MS2 scans with matching precursor matches (within the provided MS1 mass tolerance) in a series of MS2 scans between MS1 scans, using the MS1 scans as boundaries. The scan pairing utility is called immediately prior to the O-Pair C# executable in FragPipe. The workflow also supports alternative modes where the electron-based activation scan precedes the collisional scan and single scan (i.e., not paired) EThcD or EAD [32] data. Thus, a complete paired scan analysis workflow in FragPipe involves MSFragger search, PSM validation and FDR control in Philosopher, scan pairing, O-Pair search, and finally quantification, if specified (Fig. 1).

### Search parameters

Detailed parameter settings for each analysis can be found in the FragPipe workflow files in the Zenodo results repository (see “Data availability”). The workflow files are human-readable text files that can also be loaded into FragPipe to repeat the analysis with the exact parameters used here. Briefly, the search settings are as follows. For FragPipe searches, raw files were converted to mzML format using MSConvert [33] (v3.0.22068-192b762). All FragPipe (v20.0) searches used labile mode MSFragger (v3.8) search with glycans specified as mass offsets restricted to serine or threonine residues and oxonium ion filtering enabled with default ions and settings [18, 34]. All searches except the 4 mucin dataset used the full human proteome (downloaded 2019–08-22, 20464 sequences total) with common contaminants and decoys appended in Philosopher [35]. Glycan searches used either 12 or 32 composition human O-glycan databases, as indicated in the figures (Electronic Supplementary Material Tables S1, S2). All searches used semi-tryptic cleavage with a maximum of 2 missed cleavages, and the 4-mucin search additionally used StcE cleavage, cutting N-terminal to glycosylated serine or threonine, with a maximum of 10 missed cleavages to account for non-glycosylated residues [22]. Note that the 10 “missed” cleavages specified for digestion (prior to search) do not necessarily represent true missed cleavages, as not all serines and threonines are glycosylated. Precursor and product m/z tolerances were 30 and 10 ppm, respectively, with variable modifications of oxidation (M, max 3), protein N-terminal acetylation, and deamidation (N or Q, max 1), and MSFragger’s internal deisotoping, neutral loss removal, and calibration were used [19, 36]. PSM, peptide, and protein level FDR filtering were performed in Philosopher [35] (v5.0.0) using PeptideProphet [37] and ProteinProphet [38], as described previously [18]. O-Pair localization was enabled for all searches with HCD and ETD for the first and second activation types, respectively. Oxonium filtering in O-Pair was enabled for IMHO search only, using a minimum summed relative abundance of 5% and the following residue-ion pairs: HexNAc(1)Hex(1) – 366.1395^+^; Hex(1) – 163.0601^+^, 145.0495^+^; NeuAc(1) – 274.0921^+^, 292.1027^+^; NeuGc(1) – 290.0870^+^, 308.0976^+^. For TMT-labeled data in the GALNT knockout data, TMT was specified as a fixed modification on lysine and peptide N-termini and TMT quantification was performed with TMT-Integrator. Volcano plots were generated in Perseus [39] directly from the TMT-Integrator output tables. Searches were performed on a Linux server with two Intel Xeon E5-2690 CPUs (14 cores each) and 512 GB RAM available.

**Fig. 2:**
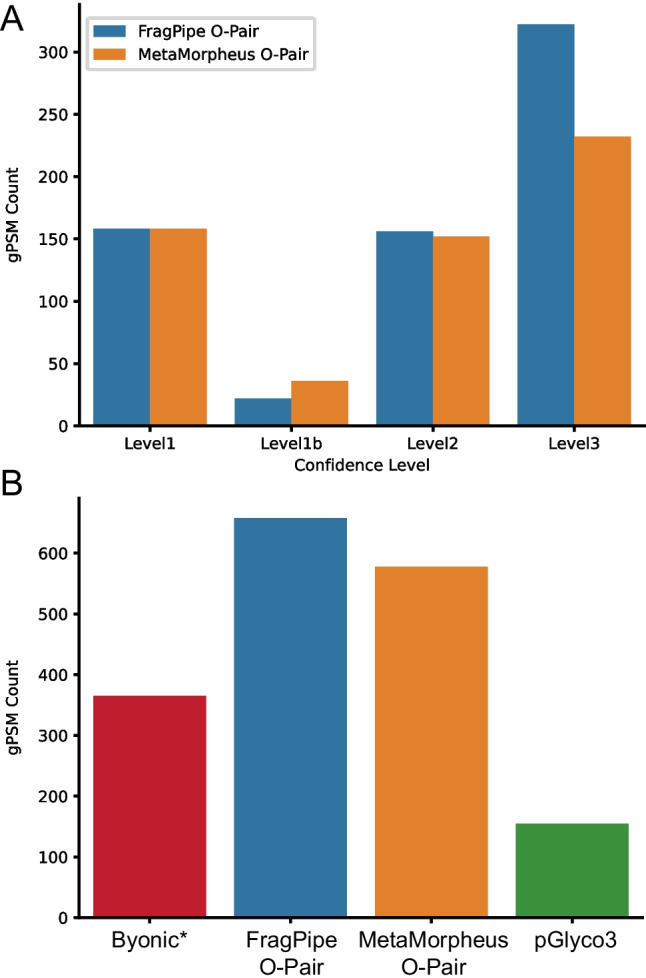
Comparison of 4-protein mixture O-glycoPSMs. **A** Total gPSMs identified by each software tool. *Byonic results are taken from Lu et al. [20] and allow a maximum of 3 glycans per peptide. All other tools allow a maximum of 5 glycans. **B** Comparison of FragPipe and MetaMorpheus O-Pair gPSMs by localization confidence level, indicating that the increase in FragPipe gPSMs is driven by Level 3 gPSMs

MetaMorpheus (v1.0.2) searches were performed with the same parameters and Linux server as used in FragPipe searches for the associated datasets, with calibration performed using the calibration task. Searches were performed using.raw files after observing lower quality results from converted mzML files. Keep topN candidates was set to 10 for all searches [22]. pGlyco3 (build20210615) was run with the same parameters as FragPipe searches, except that it does not require a maximum glycans per peptide parameter. pGlyco3 searches were also searched directly from.raw files. Searches were performed on a Windows workstation (Intel i7-8700 CPU with 12 threads, 32 GB RAM), as pGlyco3 does not support Linux. The Byonic search results shown in Fig. 2 were obtained from Lu et al*. *[20], with the following differences in parameters from the other searches shown: precursor and product ion tolerances were 10 and 20 ppm, respectively, only 3 glycans were allowed per peptide (all 12 glycans in the database were specified as common3 modifications), and the results were filtered by requiring a Byonic score greater than 200, a log_Prob_ value greater than or equal to 2, and a minimum peptide length of 4 residues.

## Results

We have previously shown that MSFragger’s peptide-first search method can greatly increase the number of collisional activation glycopeptide spectra identified compared to conventional search methods [18]. The dissociation of O-glycans from the peptide during collisional activation enables this fast and highly sensitive search, but precludes localization of the glycan(s) and even determination of the number of glycans present [7, 9, 20]. O-Pair search, implemented in MetaMorpheus [20, 21], provided an elegant solution to this problem in paired scan data, using a collision activation scan to identify the peptide and total glycan mass, and a paired electron-based activation scan to deconvolute and localize the glycan(s). To provide this capability for MSFragger-Glyco searches, we sought to incorporate the O-Pair method into FragPipe. Rather than re-implement the O-Pair search from scratch, we extracted a standalone C# executable containing the core O-Pair search algorithm and adapted it for use with FragPipe-format input and output files. The resulting FragPipe O-Pair workflow uses MSFragger search, peptide validation and FDR control in Philosopher, O-Pair localization, and downstream quantification if specified (Fig. 1).

While the core localization algorithm remains largely unchanged in the FragPipe implementation of O-Pair search, differences in MSFragger search as compared to MetaMorpheus search meant that we observed differences in the workflow output between the methods. In all MSFragger searches conducted here, we used an HCD-only scan filter so that only collisional activation scans were searched for peptide identification, and all other scans were ignored prior to O-Pair, whereas in MetaMorpheus and pGlyco3, both collisional and electron-based activation scans are used for peptide identification. However, it is possible to search electron-based activation scans as well in MSFragger, enabling, for example, the use of this workflow with pure EThcD data without paired scans (Electronic Supplementary Material Figure S1). While one might expect searching both sets of scans to identify more glycopeptides, in practice, we observed the opposite effect (Fig. 2A). Because glycans are retained intact in electron activation scans, search methods are limited to considering relatively few glycans (typically 1 or 2) to avoid a combinatorial explosion of possible localizations (Table 1). In glycopeptides with multiple glycosylation sites, search methods for the electron activation scans are thus greatly restricted, explaining why searching these scans did not increase the number of glycopeptides identified.

We first benchmarked the FragPipe O-glycopeptide analysis workflow with O-Pair on the protein mixture dataset originally used to evaluate MetaMorpheus O-Pair. The data is HCD(NCE 35)-pd-EThcD(NCE 25) activation of four recombinantly expressed mucin domain-containing proteins mixed and analyzed together. The original O-Pair defined 4 confidence levels for results: level 1, meaning all glycans are localized with probability > 0.75; level 1b, meaning some or all glycans are localized by process of elimination rather than spectral evidence; level 2, meaning at least one glycan is confidently localized but other(s) are not; and level 3, meaning no glycans are confidently localized. There was no difference in level 1 glycopeptide-spectrum matches (gPSMs) between FragPipe and MetaMorpheus O-Pair searches (Fig. 2A), as the high-quality spectra were confidently identified by both searches and identically localized by O-Pair. However, the FragPipe search identified more level 3 gPSMs than MetaMorpheus (Fig. 2A). We attribute this to the HCD-only search performed in MSFragger, allowing scan pairs with a moderate quality HCD scan and low-quality EthcD scan to be identified in the HCD-only search, but not the combined HCD and EthcD search of MetaMorpheus. Example annotated spectra of shared (Figures S2 and S3) and FragPipe-unique (Figure S4) identifications can be found in the Electronic Supplementary Material.

Expanding this comparison to include Byonic [40] and pGlyco3 [23] shows that the peptide-first search method, used by both MSFragger and MetaMorpheus, outperformed other methods in this dataset. The Byonic search results are taken from Lu et al. [20] and show the limitations of conventional searches for O-glycoproteomics. The search considered a maximum of three O-glycans per peptide as allowing additional glycans resulted in prohibitively long search times, whereas all other search tools allowed five glycans per peptide. As a result, the Byonic search identified fewer gPSMs than FragPipe or MetaMorpheus (Fig. 2B) and took many hours to do so [20], as opposed to 1 min or less for all other tools. pGlyco3 also identified fewer gPSMs than other tools in this dataset (Fig. 2B), likely due to its requirement to observe at least one Y ion (intact peptide backbone with a partial glycan attached) to identify a glycopeptide. Due to the high activation energy employed in this dataset, very few such ions were present (Electronic Supplementary Material Figure S5), resulting in lower performance than the peptide-first methods in this case.

**Fig. 3:**
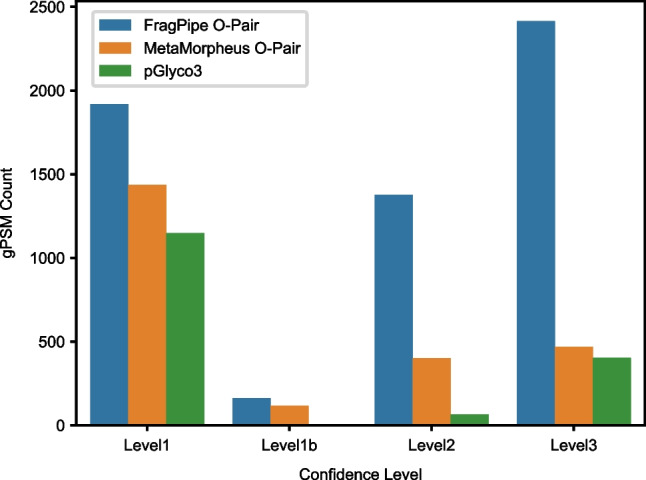
Comparison of urine O-glycoproteomics results by localization confidence level

We next sought to use the speed of MSFragger search to enable O-glycoproteomic analysis of entire proteomes, as opposed to individual proteins. With millions of possible peptides, or billions if considering semi-enzymatic peptides and/or other variable modifications, search speed becomes essential to analyzing whole glycoproteome data. Conventional search methods, which additionally have to contend with up to millions of configurations of glycans on a single glycopeptide (Table 1), are generally incapable of analyzing proteome scale data unless glycosylation is restricted to just a few glycans. Hence, we did not consider any conventional searches in the whole proteome analyses, as the search time would have been prohibitive. Comparing FragPipe, MetaMorpheus, and pGlyco3 on a dataset of O-glycopeptides from human urine [41], we observe similar trends to the protein standards dataset. The peptide-first searches of MSFragger and MetaMorpheus identified more glycopeptides than the glycan-first search of pGlyco3, but to a lesser extent than in the protein standards dataset as the collision energies employed in this dataset were much lower (Fig. 3). In addition, the HCD-only search of MSFragger identified many more level 2 and level 3 gPSMs than MetaMorpheus or pGlyco3, to a much greater extent than in the mucin standards data. We attribute this to the generally lower quality of EThcD spectra in this older dataset, taken with much lower supplemental activation energy, which resulted in much lower scores for combined HCD and EThcD pairs than HCD scans alone. While we do not anticipate most datasets to exhibit such a difference in quality between HCD and EThcD scans, it does illustrate the value of being able to perform an HCD-only search in such cases. Furthermore, the MSFragger average run time was only about 2 min per raw file (Table 2). Finally, we note that the samples in this analysis were not treated with PNGase F to remove N-glycans, unlike all other datasets considered. N-glycans are not explicitly considered by the search method but can be matched when the sum of O-glycan masses matches that of an N-glycan. In this case, less than 2% of glycoPSMs contained sufficient residues to form the trimannosyl chitobiose N-glycan core and the majority, but not all, of these 2% had evidence indicating O-glycosylation rather than N-. However, PNGase F treatment is strongly recommended when using this method to avoid potential misidentifications.

**Table 2:** Analysis time comparison for complete search of urine O-glycoproteomics dataset. *Note that pGlyco3 search was run on a different computer than FragPipe and MetaMorpheus (see “Methods”) and did not use parallelization due to computer memory constraints

	Glycan database size	Max glycans	Time per file (min)
FragPipe	12	5	2.0
MetaMorpheus	12	5	204.0
pGlyco3	12	n/a	54.6*

**Fig. 4:**
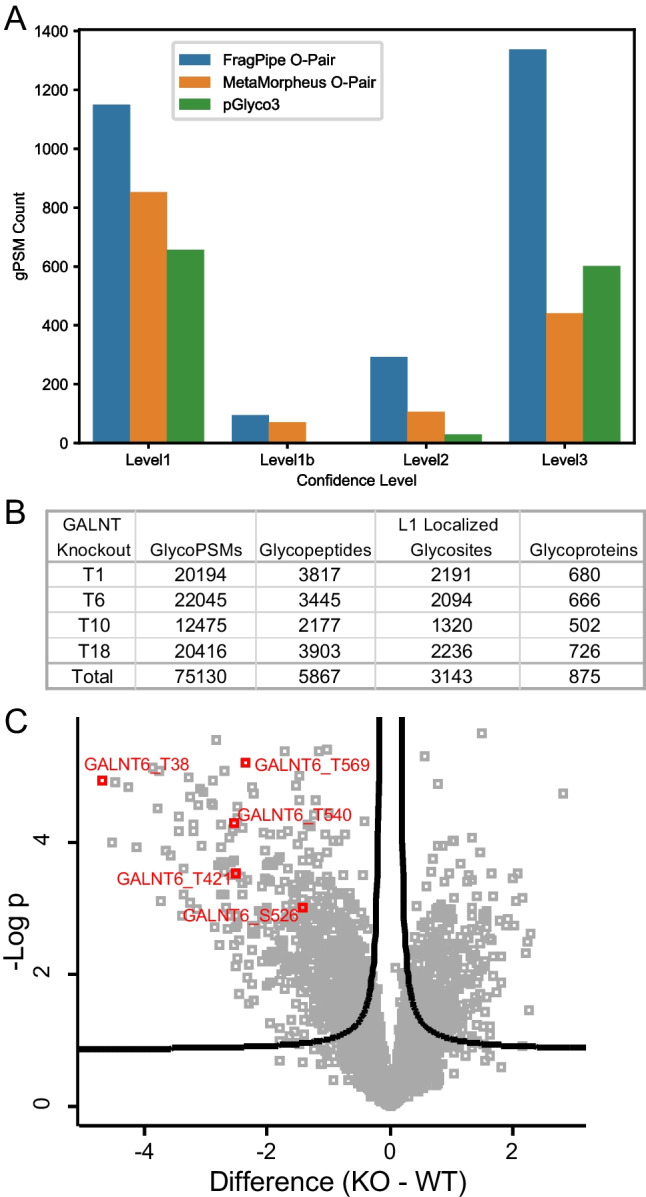
Comparison of human skin cell line O-glycoproteomics results. **A** gPSMs identified by confidence level for each software tool in one LC–MS file, using a database of 32 glycans. **B** Glyco PSMs, peptides, sites, and proteins identified by FragPipe O-Pair search in all 8 fractions of 4 knockouts (32 LC–MS files total). **C** Example volcano plot of quantified glycosites from GALNT-6 knockout data. Detected GALNT6 glycosites are highlighted as an example, showing large reductions in abundance following knockout

As O-glycoproteomics experiments increase in scale, there is a need for software capable of searching and quantifying O-glycopeptides proteome-wide in larger datasets. To demonstrate this capability with FragPipe, we analyzed a recently published dataset that used TMT labeling to investigate GALNT-knockout cell lines [30]. Our FragPipe O-linked TMT workflow took 38.5 min to analyze 32 raw files (4 TMT experiments with 8 fractions each) or 12.5 min to analyze a single file (Table 3), including search, validation, localization with O-pair, and quantification of all peptides and glycopeptides. We could not run pGlyco3 on the same Linux server that was used for FragPipe and MetaMorpheus as it supports Windows only, and while it can use multiple processes to analyze multiple data files in parallel, in practice, we found this exceeded the 32 GB of RAM available on the Windows machine used for testing. As a result, we show a time of 37.9 min for pGlyco3 to analyze one raw file, but this would be faster if a Windows computer with much more RAM was used. As in the urine dataset, MSFragger search identified more gPSMs than MetaMorpheus or pGlyco3, predominantly level 3 identifications with low-quality EThcD scans (Fig. 4A). In total from 4 knockout samples, FragPipe identified over 75,000 gPSMs from 3,143 confidently localized glycosites on 875 glycoproteins (Fig. 4B). Compared to the original analysis, performed using SEQUEST and only considering two glycans vs the 32 considered in FragPipe, we identified nearly 600 more glycosites. This is due both to the increased sensitivity of the O-Pair style search vs conventional search and the much larger glycan database, allowing for identification of sites bearing only sialylated glycans, for example. The O-Pair glycan localization results were taken directly into TMT-Integrator to generate site-specific quantitative comparisons automatically as part of the FragPipe workflow. An example volcano plot comparing the glycosites quantified in GALNT6 knockout vs wild-type cell lines is shown in Fig. 4C. TMT-Integrator also produces reports at the glycoform (i.e., glycosite-glycan combination), glycopeptide, and glycoprotein levels to support a wide variety of glycoproteomics analyses.

**Table 3:** Analysis time comparison for Human skin O-glycoproteomics dataset. *Note that pGlyco3 search was run on a different computer than FragPipe and MetaMorpheus (see “Methods”)

	Glycan database size	LC–MS files analyzed	Total time (min)
FragPipe	32	1	12.5
MetaMorpheus	32	1	1207.5
pGlyco3	32	1	37.9*
FragPipe	32	32	38.5

**Fig. 5:**
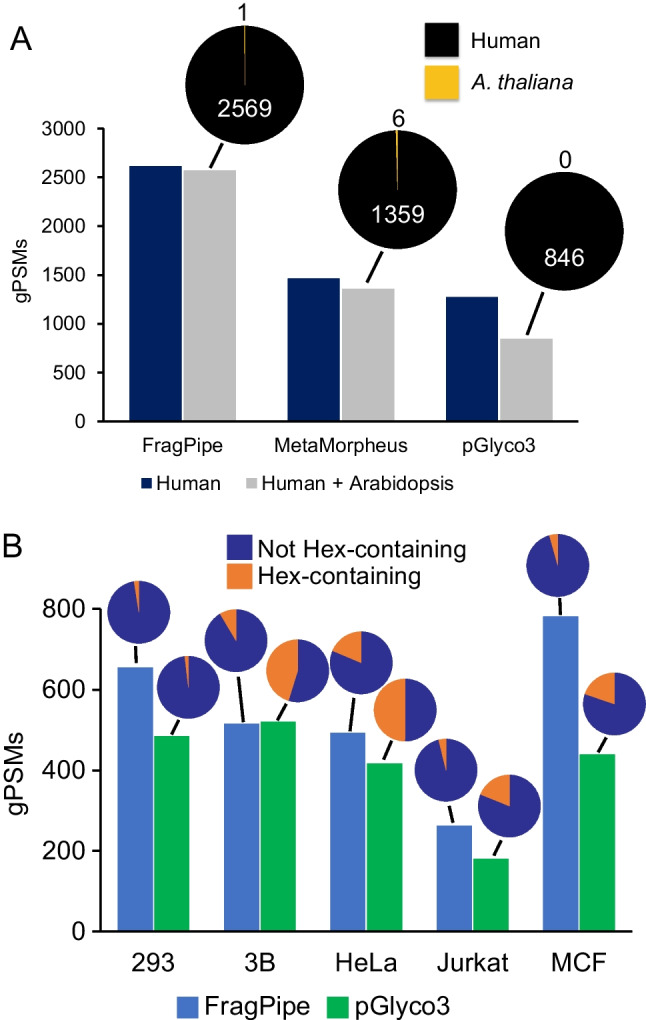
Entrapment protein and glycan searches. **A** Total gPSMs identified for searches of human skin cell line data using human-only (blue bars) or human plus *A. thaliana* protein databases (gray bars). Pie charts indicate the numbers of human (blue) or *A. thaliana* (yellow) gPSMs reported at 1% FDR. **B** Glycan entrapment search of IMHO-treated cell lines. Hexose-containing glycans are expected only at low levels due to IMHO treatment. Bars show gPSMs identified after oxonium ion filtering (FragPipe) or at 1% glycan FDR (pGlyco3). Pie charts show the proportion of gPSMs with hexose (orange) and non-hexose (blue) containing glycan(s) for each search

Finally, controlling the false discovery rate effectively is essential for moving to automated glycopeptide analysis methods and can be challenging to do in complex glycopeptide searches. We performed two entrapment experiments to validate the FDR control of our FragPipe workflow and compare to other software tools for O-glycoproteomics analysis. First, we searched a combined human (20,464 sequences) and Arabidopsis thaliana (27,618 sequences) proteome to assess the peptide-level FDR. In all three software tools, the number of *A. thaliana* PSMs reported remained well under the set 1% FDR, with a single entrapment PSM from FragPipe (0.04% entrapment), 6 from MetaMorpheus (0.4%), and 0 from pGlyco3 (0%) (Fig. 5A). While it did not report any entrapment gPSMs, pGlyco3 experienced a 34% reduction in human gPSMs identified when searching the combined database vs human alone, much more than the 1.8% and 7.1% reductions for FragPipe and MetaMorpheus, respectively. These well-controlled entrapment rates are very encouraging given the large search space of the semi-enzymatic search considering 32 glycans with up to 5 glycans per peptide. Second, we evaluated FragPipe using the glycan entrapment experiment introduced by pGlyco3. In this experiment, various human cell lines were treated with an O-glycan elongation inhibitor to prevent the addition of galactose to the initial O-GalNAc [23]. Thus, glycans containing hexose are expected to be present only at low levels, in contrast to typical O-glycans that frequently contain hexose(s), such as the core 1 glycan GalNAc-Gal. We added an oxonium ion filtering step to our FragPipe O-Pair implementation, allowing user-specified oxonium ion(s) to be required in a spectrum to consider glycans containing defined monosaccharides. In this case, we required the 145.05 ([Hex-H_2_O]^+^), 163.06 (Hex^+^), and 366.14 (HexNAc-Hex^+^) ions to be found for hexose-containing glycans, as well as sialic acid-specific ions for sialic acid-containing glycans. In each cell line, we obtained as many or more gPSMs with FragPipe compared to pGlyco3, while also more stringently filtering out hexose-containing gPSMs (Fig. 5B). This oxonium ion filtering is not a glycan composition FDR filter and does not guarantee that the correct glycan compositions are reported; however, it does provide a useful and customizable tool for improving the quality of automated glycan identifications.

## Conclusions

We have developed a site-specific, quantitative analysis pipeline for O-glycoproteomics data in FragPipe that is capable of extremely fast identification and quantification of glycopeptides across the entire proteome. The paired-scan localization method of O-Pair is the key to enabling site-specific O-glycopeptide analysis with this speed and scale. Integrating the O-Pair method into FragPipe allows users to leverage FragPipe’s many capabilities for O-glycoproteomics, particularly including quantitation tools for both label-free and isobarically labeled data. Combined with the many other capabilities of FragPipe, this enables quantitative analysis of large-scale O-glycoproteomics datasets to be accomplished in minutes, including searches for full proteomes and glycomes that are infeasible with conventional search methods. The O-Pair localization algorithm is available in FragPipe 20.0 + and is included as part of a built-in workflow template for O-linked glycoproteomics data. While much of the analysis can be automated with FragPipe, validation of lower confidence localizations and glycan identifications remains important. In future development, we hope to combine glycan composition assessments and O-Pair localization confidence to provide true O-glycopeptide FDR control.

## Supplementary Information

Below is the link to the electronic supplementary material.

Supplementary file1 (DOCX 388 KB)

## Data Availability

The raw mass spectrometry files analyzed as part of this study are available from the following public ProteomeXchange repositories (see “Methods” for details): PXD017646, MSV000083070, PXD036791, and MSV00008677. Processed search results and search parameter files generated in this study have been deposited to Zenodo with 10.5281/zenodo.11223818 and can be accessed at https://zenodo.org/records/11223818.
