# Supplementary material for: Quantitative proteome-wide O-glycoproteomics analysis with FragPipe
Source: Anal Bioanal Chem. 2024 Jun 15;417(5):921–30. doi: 10.1007/s00216-024-05382-x (PMC11648966; doi:10.1007/s00216-024-05382-x)
Supplement: Supplementary file 1 — Supplementary file1 (DOCX 388 KB) [file 216_2024_5382_MOESM1_ESM.docx]

Supporting Information for

Quantitative Proteome-wide O-Glycoproteomics Analysis with FragPipe

Daniel A. Polasky^1^, Lei Lu^2,3^, Fengchao Yu^1^, Kai Li^4^, Michael R. Shortreed^2^, Lloyd M. Smith^2^, and Alexey I. Nesvizhskii^1,4^

^1^Department of Pathology, University of Michigan

^2^Department of Chemistry, University of Wisconsin-Madison

^3^Department of Pharmaceutical Chemistry, University of San Francisco

^4^Department of Computational Medicine and Bioinformatics, University of Michigan

Contents

1. Table S1. 12 glycan database used for searches.
2. Table S2. 32 glycan database used for searches.
3. Figure S1. Analysis of EThcD-only data with FragPipe
4. Figure S2. Example of glycopeptide identification via paired scan search in FragPipe.
5. Figure S3. MetaMorpheus annotation of the same scan pair as in Figure S3.
6. Figure S4. Example of a Level 3 glycopeptide identified only by FragPipe.
7. Figure S5. Comparison of Y ion frequencies in mucin and urine datasets.

Supplementary Table 1. 12 glycan database used for searches.

| Composition | Mass |
| --- | --- |
| HexNAc(1) | 203.0794 |
| HexNAc(1)Hex(1) | 365.1322 |
| HexNAc(1)NeuAc(1) | 494.175 |
| HexNAc(2)Hex(1) | 568.2116 |
| HexNAc(1)Hex(1)NeuAc(1) | 656.2276 |
| HexNAc(1)Hex(1)NeuAc(2) | 947.323 |
| HexNAc(2)Hex(2)NeuAc(1) | 1021.36 |
| HexNAc(2)Hex(2)NeuAc(2) | 1312.455 |
| HexNAc(2)Hex(2) | 730.2644 |
| HexNAc(2)Hex(1)NeuAc(1) | 859.307 |
| HexNAc(2)Hex(2)Fuc(1)NeuAc(1) | 1167.418 |
| HexNAc(2)Hex(2)Fuc(1)NeuAc(2) | 1458.513 |

Supplementary Table 2. 32 glycan database used for searches.

| Composition | Mass |
| --- | --- |
| HexNAc(1) | 203.0794 |
| HexNAc(1)Hex(1) | 365.1322 |
| HexNAc(2) | 406.1588 |
| HexNAc(1)NeuAc(1) | 494.1748 |
| HexNAc(1)Hex(1)Fuc(1) | 511.1901 |
| HexNAc(1)Hex(2) | 527.185 |
| HexNAc(2)Hex(1) | 568.2116 |
| HexNAc(1)Hex(1)NeuAc(1) | 656.2276 |
| HexNAc(1)Hex(2)Fuc(1) | 673.2429 |
| HexNAc(2)Hex(1)Fuc(1) | 714.2695 |
| HexNAc(2)Hex(2) | 730.2644 |
| HexNAc(1)Hex(1)Fuc(1)NeuAc(1) | 802.2855 |
| HexNAc(1)Hex(2)NeuAc(1) | 818.2804 |
| HexNAc(2)Hex(1)NeuAc(1) | 859.307 |
| HexNAc(2)Hex(1)Fuc(2) | 860.3274 |
| HexNAc(2)Hex(2)Fuc(1) | 876.3223 |
| HexNAc(1)Hex(1)NeuAc(2) | 947.323 |
| HexNAc(1)Hex(2)Fuc(1)NeuAc(1) | 964.3383 |
| HexNAc(2)Hex(1)Fuc(1)NeuAc(1) | 1005.365 |
| HexNAc(2)Hex(2)NeuAc(1) | 1021.36 |
| HexNAc(2)Hex(2)Fuc(2) | 1022.38 |
| HexNAc(3)Hex(3) | 1095.397 |
| HexNAc(1)Hex(2)NeuAc(2) | 1109.376 |
| HexNAc(2)Hex(2)Fuc(1)NeuAc(1) | 1167.418 |
| HexNAc(1)Hex(1)NeuAc(3) | 1238.418 |
| HexNAc(3)Hex(3)Fuc(1) | 1241.455 |
| HexNAc(2)Hex(2)NeuAc(2) | 1312.455 |
| HexNAc(2)Hex(2)Fuc(2)NeuAc(1) | 1313.476 |
| HexNAc(3)Hex(3)NeuAc(1) | 1386.492 |
| HexNAc(3)Hex(3)Fuc(2) | 1387.512 |
| HexNAc(2)Hex(1)NeuAc(3) | 1441.498 |
| HexNAc(2)Hex(2)Fuc(1)NeuAc(2) | 1458.513 |

Supplementary Figure 1. Analysis of EThcD-only data with FragPipe. NCE values are listed in parentheses for each activation or supplemental activation. Top: MSFragger search was performed on EThcD scans, considering *b, y, c, z,* and Y ions, and the 12 glycan database (max 5 per peptide) set as mass offsets. Note that this precludes identifying *c* or *z* ions with intact glycans from multiply glycosylated peptides (but avoids the associated combinatorial explosion of peptide candidates). Multiply glycosylated peptides rely on *b* and *y* ions for identification by MSFragger, which is likely the reason the number of PSMs increases consistently with increasing supplemental activation. O-Pair search was performed using *c* and *z* ions, with the “single-scan” option set, which treats each scan as its own pair (i.e., the MSFragger result for a scan is passed to O-Pair, and O-Pair is told to localize the glycans using that same scan). Compared to paired scan analysis of the same sample (bottom graph), EThcD only analysis yielded fewer total PSMs, but a higher proportion of Level 1 PSMs. This is expected, as the glycopeptides that generate high quality EThcD spectra for identification should generally have sufficient information to localize the glycan(s), while spectra that can be identified only from HCD are less likely to have sufficient information in the EThcD scan to confidently localize glycan(s).


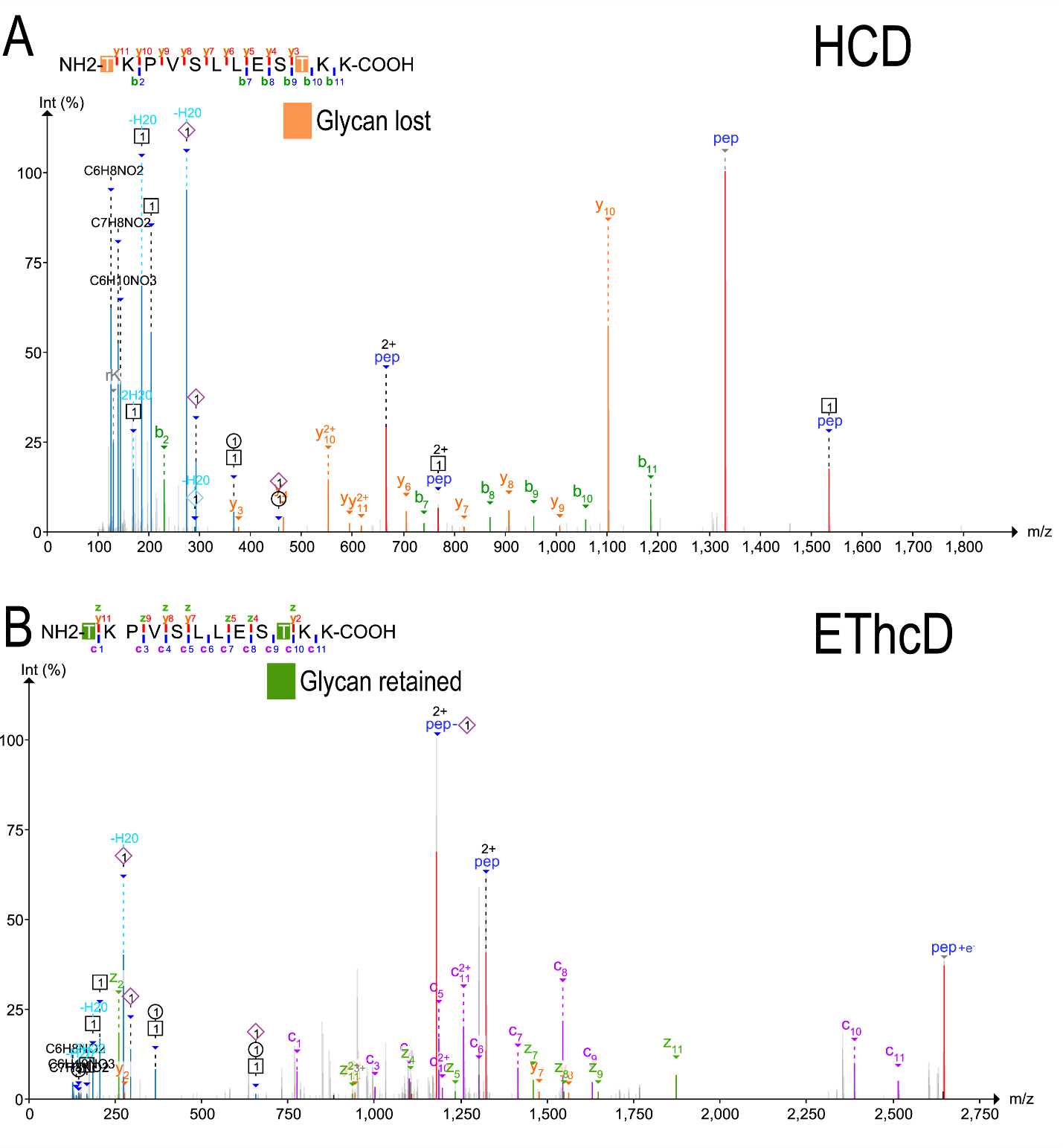


Supplementary Figure 2. Example of glycopeptide identification via paired scan search in FragPipe. A) HCD spectrum, with *b* and *y* ions annotated after complete loss of glycans. Oxonium ions (blue) and glycopeptide Y ions (red) are also shown. Note that if glycans are left intact (as in a conventional search) no *b* and *y* ions are matched at all. The HCD spectrum confidently identifies the peptide sequence and total glycan mass. B) Paired EThcD spectrum annotated with glycan locations determined by O-Pair (HexNAc(1)Hex(1)NeuAc(1) at both T1 and T10). Intact glycans on *c* and *z* fragment ions allow confident localization of these glycan masses at these sites.


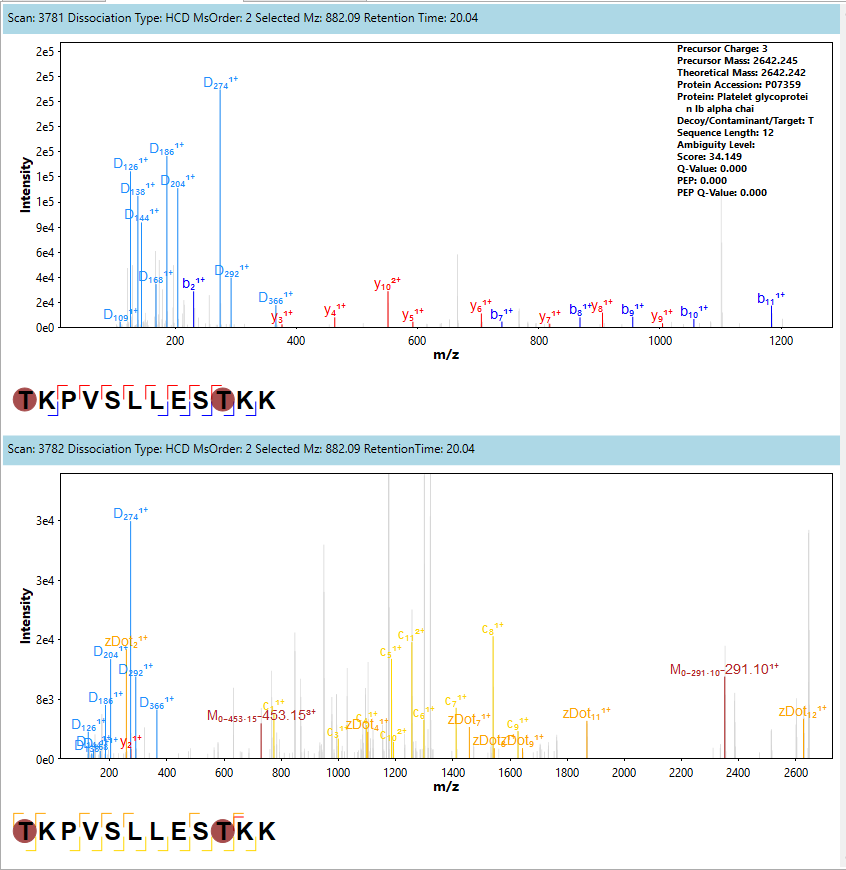


Supplementary Figure 3. MetaMorpheus spectrum annotation of the same paired scans as in Supplementary Figure 2. Top: HCD spectrum showing oxonium ions (light blue) and peptide backbone *b* (blue) and *y* (red) fragments. Bottom: paired EThcD spectrum showing *c* (yellow) and *z* (orange) fragments in addition to oxonium ions.


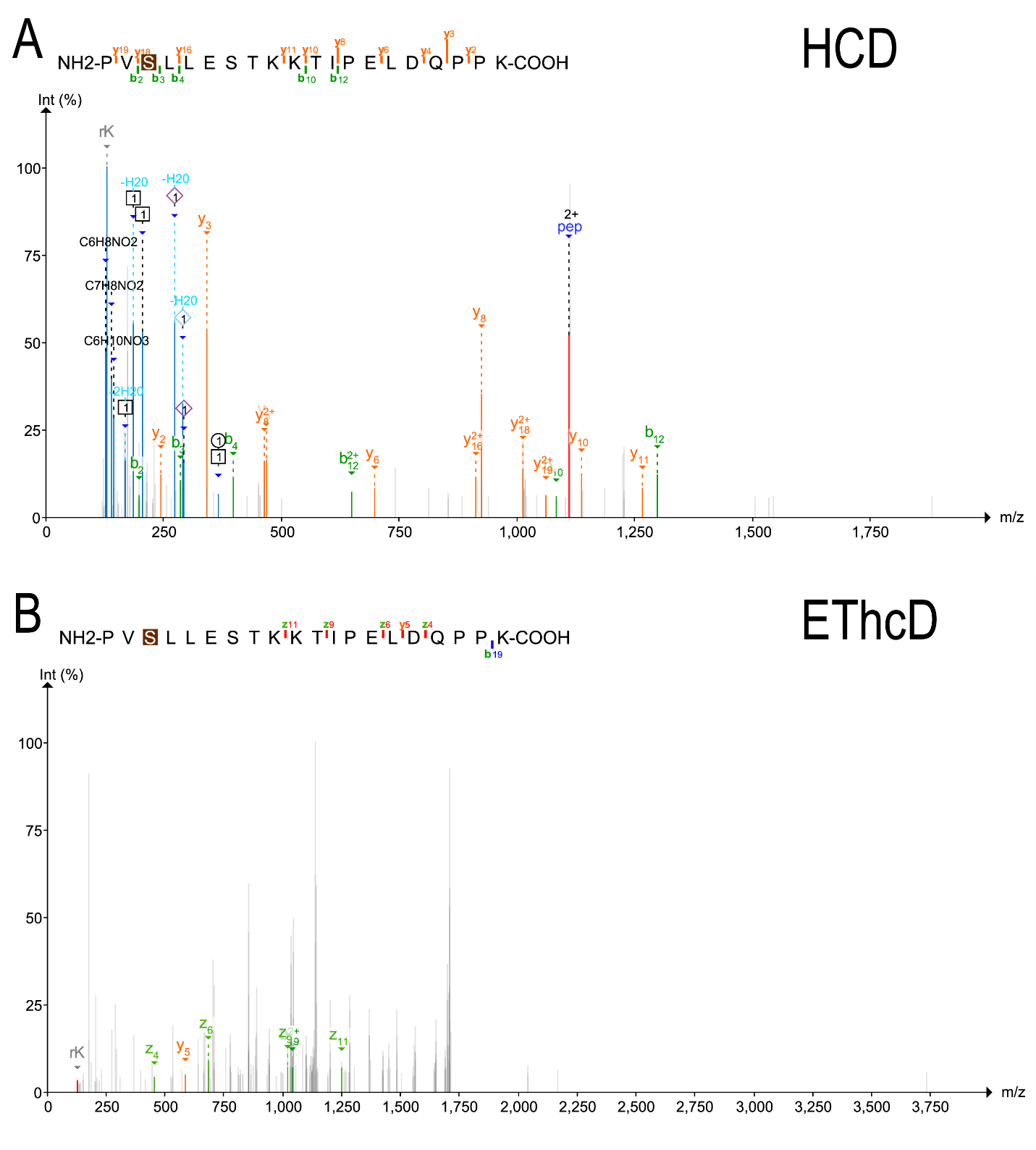


Supplementary Figure 4. Example paired spectrum set identified by FragPipe but not other tools. A) HCD spectrum with a clear match to the peptide sequence and good score. B) EThcD spectrum showing very few fragment ions matched. FragPipe reported this as a Level 3 identification based on the HCD spectrum alone, whereas other tools did not report it as a match, presumably due to the lack of ions matched in the EThcD spectrum.

Supplementary Figure 5. Comparison of Y ion frequencies in 4 mucin mixture and human urine O-glycopeptide datasets. Y ions are specified by composition (N=HexNAc, H=Hexose) in increasing order of size. The urine dataset exhibits considerably more frequent Y ions than the mucin dataset, likely a result of the lower normalized collision energies used.
